# Identification of tumor initiating cells and early marker genes in normal colonic epithelium that lead to neoplastic transformation

**DOI:** 10.21203/rs.3.rs-7914753/v1

**Published:** 2025-10-27

**Authors:** Sangeeta Jaiswal, Stephanie The, Tse-Shao Chang, Jiaqi Shi, Thomas D Wang

**Affiliations:** 1. Division of Gastroenterology, Department of Internal Medicine, University of Michigan, Ann Arbor, Michigan, USA.; 2. Cancer Data Science Shared Resource, Department of Biostatistics, University of Michigan School of Public Health, Ann Arbor, Michigan, USA; 3. Department of Mechanical Engineering, University of Michigan, Ann Arbor, Michigan, USA.; 4. Department of Pathology, University of Michigan, Ann Arbor, Michigan, USA.; 5. Department of Biomedical Engineering, University of Michigan, Ann Arbor, Michigan, USA

**Keywords:** tumor-initiating cells, colorectal cancer, single-cell RNA-seq, epithelial-to-mesenchymal transition, *ETS2*, *SOD3*, *GPRC5A*

## Abstract

**Background & Aims:**

Colorectal cancer (CRC) remains a major cause of cancer-related morbidity and mortality worldwide. Although the adenoma-carcinoma sequence and associated genetic alterations are well characterized, the earliest cellular and molecular events that initiate tumorigenesis within histologically normal colonic epithelium remain poorly defined. This study aims to identify tumor-initiating cells (TICs) and early transcriptional markers of neoplastic transformation using single-cell RNA sequencing (scRNA-seq) from paired normal-appearing and transformed human colonic tissues.

**Methods:**

Fresh biopsies from histologically normal-appearing colonic mucosa and paired polyps, including tubular adenomas, sessile serrated adenomas, and adenocarcinoma, were collected from 7 human subjects. Single-cell transcriptomes were generated using the 10X Genomics platform and analyzed with Seurat, Monocle 2, CytoTRACE, GSVA, GSEA, RNA velocity, and InferCNV. Tumor-associated states were inferred utilizing clustering, trajectory analysis, pathway enrichment, copy number variation profiling, and validated spatially by RNA-FISH.

**Results:**

A total of 51,054 high-quality single-cell transcriptomes were resolved into 33 epithelial and stromal clusters. Tumor-specific stem-like (tSTM, cluster 0) and deep crypt secretory (tDCS, cluster 10) populations were enriched in adenomas, whereas sub-clustering of tSTM identified TICs (subclusters 4 and 6) derived largely from histologically normal mucosa. TICs exhibited strong stemness potential, genomic instability, and early activation of epithelial-mesenchymal transition (EMT) and interferon signaling, accompanied by suppression of oxidative phosphorylation. *ETS2*, *SLC12A2*, and *LEFTY1* were identified as TIC-specific markers, while *SOD3* and *GPRC5A* showed progressive upregulation along the TIC-to-tSTM trajectory. RNA-FISH confirmed spatial expression of candidate genes in adenomatous crypts, and independent validation using the COLON MAP dataset supported the presence and diagnostic performance of TIC-associated markers.

**Conclusions:**

This study identifies TICs as the developmental origin of neoplastic stem-like states and delineates early transcriptional and pathway reprogramming events that drive the transition from normal to premalignant colonic epithelium. These findings provide new insight into CRC initiation and nominate biomarkers with translational potential for early detection and therapeutic targeting.

## 1. Introduction

Colorectal cancer (CRC) contributes substantially to the worldwide health care burden. Globally, over 1.9 million cases are diagnosed each year leading to more than 900,000 deaths annually [1]. In the U.S., about 152,810 people are diagnosed yearly, and annual mortality is about 53,010 [2]. The adenoma-carcinoma sequence has been widely accepted as the underlying molecular process that leads to sporadic CRC development [3]. A series of genetic mutations occur in normal colonic mucosa that result in spontaneous formation of adenomas followed by invasive cancer [4]. Inactivation of tumor suppressor genes, such as *APC*, leads to dysregulated WNT signaling [5], and activation of oncogenes, such as *KRAS*, stimulates the MAPK pathway [6]. Sequential genetic and epigenetic changes over time then drive proliferative changes that may result in adenocarcinoma [7]. Thus, new approaches to detect CRC at an early stage, when treatment options are more effective, are urgently needed. Such approaches may be enabled by defining precursor cell populations, identifying early marker genes, and clarifying molecular pathways that initiate malignant transformation.

Previously, bulk RNA sequencing methods have been used primarily to investigate CRC molecular genetics [8]. Transcriptome profiling, biomarker discovery, cancer heterogeneity characterization, and investigation of therapeutic resistance mechanisms have been investigated using mucosal tissues. However, only average gene expression levels across diverse cell populations are measured [9]. Recently, cancer stem cells (CSCs) have been implicated to play a key role in CRC initiation and growth [10–12]. These self-renewable, pluripotent cells have an innate capacity to regenerate as well as initiate tumors. Marker genes for CSCs in CRC have been reported, and include *CD44*, *CD133*, *LGR5*, *DCLK1*, *CD166*, *CD26*, and *CD24* [13–18]. Single cell RNA sequencing (scRNA-seq) is an emerging approach that provides gene expression at the level of individual cells and can provide a more detailed analysis of cellular diversity [19–21]. This approach can be used to identify rare cell subpopulations, such as CSCs, subtle transcriptional variations, and temporal gene expression dynamics [22,23]. This method provides an opportunity to distinguish marker genes in premalignant versus malignant epithelium that may drive cancer initiation.

Tumor initiation in normal colonic epithelium that leads to cancer transformation and progression is a complex biological process that may involve a variety of precursor cells, tumor-initiating cells, and transitional cells [24–26]. scRNA-seq enables high-resolution analysis of tens of thousands of transcriptomes to characterize tumor heterogeneity in an unbiased manner and to discover novel cell states. Previous studies have used scRNA-seq to build molecular atlases, study the microenvironment, and define molecular classifications; however, few have traced CSCs within histologically normal colonic epithelium to identify the earliest molecular events that precede malignant transformation. The aim of this manuscript is to use single cell transcriptomics to identify tumor-initiating cells in normal colonic epithelium and to define the key molecular events and early transcriptional markers that drive progression to colorectal cancer. Specific epithelial cell populations from paired adenomas and adjacent normal-appearing mucosa collected fresh from human subjects at increased risk for CRC will be examined.

## 2. Methods

### Human subject and tissue collection

Fresh human colonic tissues were obtained from 7 adult patients undergoing routine screening colonoscopy at Michigan Medicine under IRB approved protocol HUM00102771 (IRBMED). Informed written consent was obtained from all participants prior to enrollment. For each subject, paired biopsies were collected from colonic polyps and adjacent normal appearing mucosa. Each specimen was deidentified prior to processing to ensure patient confidentiality. All authors had full access to the study data and approved the final version of the manuscript.

### Tissue Processing and Histological Evaluation

Biopsied colonic tissues were bisected upon collection. One portion was immediately processed for scRNA-seq to preserve transcriptomic integrity while the other was fixed in 10% neutral buffered formalin and paraffin-embedded for histological evaluation. H&E staining was performed on 5-μm tissue sections, and diagnoses were confirmed by an expert GI pathologist (JS).

### Single-Cell Suspension Preparation and Sequencing

Single-cell suspensions were generated from freshly collected colon tissues using the Neural Tissue Dissociation Kit (Miltenyi Biotec, #130-092-628) according to the manufacturer’s protocol. Following dissociation, single cells were encapsulated into nanodroplets using the Chromium Controller (10X Genomics), and single-cell libraries were constructed using the Chromium Single Cell 5’ Library & Gel Bead Kit (10X Genomics). High-throughput sequencing of the resulting libraries was performed on the NovaSeq 6000 sequencer to enable transcriptome-wide profiling at single-cell resolution.

### Data Preprocessing and Integration

Raw sequencing reads were aligned and quantified using Cell Ranger (10X Genomics) against the GRCh38 human reference genome [27]. Processed data were analyzed using Seurat v3.1.0 within the R environment [28,29]. Standard quality control filtering was applied to exclude cells with fewer than 200 detected genes or with mitochondrial gene content <25% of total UMI counts. Following quality control, data were normalized, log-transformed, and integrated across all patient samples using the Seurat integration pipeline to correct for inter-sample batch effects. Batch correction was visually validated using t-SNE plots, which confirmed effective integration of cells across sample.

### Clustering and Cell Type Annotation

Dimensionality reduction was performed using principal component analysis (PCA), with the top 18 principal components selected for downstream clustering based on variance explained. Unsupervised clustering was then applied to groups of transcriptionally distinct cell populations. Cluster identities were visualized using both UMAP and t-SNE projections to assess spatial separation. Differentially expressed genes (DEGs) for each cluster were identified using the Seurat function FindAllMarkers. Cluster annotation was guided by the expression of established canonical marker genes and led to the identification of major cell types.

### Identification of Tumor-Specific Clusters

Cell clusters were annotated based on composition across tissue types and the expression of canonical marker genes to identify tumor-associated epithelial subpopulations. Clusters enriched in polyp-derived epithelial cells were examined for differential abundance compared to clusters derived from histologically normal mucosa. Marker genes associated with intestinal stem cells, e.g. *OLFM4* and *LGR5*, and secretory lineages, e.g. *REG4* and *MUC2*, were used to classify stem-like and deep crypt secretory (DCS) cell populations. Corresponding clusters with similar marker profiles in both normal and polyp were annotated as normal stem-like (nSTM) and normal DCS (nDCS) populations. Immunofluorescence (IF) staining was performed on FFPE sections using antibodies against *OLFM4* and *REG4* to validate gene expression at the protein level. Confocal microscopy was used to image fluorescence signal and confirm spatial localization of marker expression in epithelial crypt.

### Gene Set Enrichment Analysis (GSEA) and GO Annotation

Differentially expressed genes (DEGs) between tumor-specific and normal epithelial subtypes, specifically, tumor stem-like (tSTM) versus normal stem-like (nSTM), and tumor deep crypt secretory (tDCS) versus normal DCS (nDCS) cells, were subjected to gene set enrichment analysis (GSEA) using the clusterProfiler package in R [30]. Gene sets for Hallmark pathways were obtained from the Molecular Signatures Database (MSigDB) [31–34]. DEGs with adjusted p-values <0.05 were ranked and used to compute enrichment scores. Enrichment was assessed using normalized enrichment scores (NES) and false discovery rate (FDR)-adjusted q-values. Visualization of enriched pathways was performed using dot plots to illustrate functional profiles of tumor-associated versus normal epithelial cell populations.

### CytoTRACE analysis

CytoTRACE (Cellular Trajectory Reconstruction Analysis using gene Counts and Expression) was performed to infer the differentiation potential of single cells using a pre-processed Seurat object [35]. The Seurat object, containing filtered and normalized single-cell RNA-seq data, was converted to a gene expression matrix using the as.matrix() function on the raw counts slot (9arkov_object[[“RNA”]]@counts). This matrix was used as input for the CytoTRACE() function from the CytoTRACE R package (v0.3.3). Default parameters were applied unless stated otherwise. The resulting CytoTRACE scores, which estimate cellular plasticity based on transcriptional diversity, were mapped back onto the Seurat object metadata. These scores were then visualized on UMAP embeddings to reveal differentiation gradients across clusters. Higher CytoTRACE scores indicated less differentiated, more stem-like cell states, and were used to support downstream trajectory analyses.

### InferCNV analysis

The copy number variation (CNV) score in the stem cell population cells was calculated based on the single-cell transcriptomic profiles using InferCNV (https://github.com/broadinstitute/inferCNV (ver 1.22.0). Cells from cluster 11 and 12 obtained from normal specimens were selected as references. For the inferCNV analysis, the following parameters were used: “denoise,” default hidden 9arkov model settings, and a value of 0.1 as the “cutoff” value. Finally, the subclusters with relatively higher CNV scores were considered malignant cells.

### Subclustering and Tumor-Initiating Cell (TIC) Identification

Epithelial clusters were re-clustered into transcriptionally distinct subpopulations using Seurat to investigate the transcriptional evolution of tumor-specific stem-like (tSTM) cells. Subclusters were annotated based on tissue origin, and specific subpopulations predominantly derived from histologically normal epithelium were flagged for further analysis as candidate tumor-initiating cells (TICs). Differential gene expression analysis was performed between TIC-enriched subclusters and the main tSTM population to identify early molecular events associated with tumor initiation. GSVA was subsequently conducted to examine pathway alterations associated with this transition, focusing on Hallmark gene sets. Genes showing progressive upregulation along the TIC-to-tSTM continuum were selected for further analysis to identify early transformation markers. Expression trajectories of candidate genes were visualized using pseudotime mapping.

### Pseudotime Trajectory Analysis

Trajectory inference was performed using the Monocle 2 R package to investigate transcriptional transitions during epithelial transformation [37]. For pseudotime analysis, stem cell-associated clusters were extracted. Subclusters within the tSTM population were also isolated to evaluate the potential origin and differentiation trajectories of tumor-initiating cells (TICs) to further resolve lineage dynamics. Dimensionality reduction was carried out using the DDRTree algorithm, and cells were ordered along inferred trajectories using the orderCells function. Differential gene expression across pseudotime was computed using differentialGeneTest. Principal component-based visualization was used to map transcriptional transitions across pseudotemporal space. Gene expression changes and pathway activity along trajectory components were used to characterize phenotypic shifts associated with early tumorigenesis.

### GSVA and Correlation with Pseudotime

GSVA was performed to quantify cell-level pathway activity across pseudotime trajectories with a focus on key Hallmark pathways such as EMT and oxidative phosphorylation (PHOS). GSVA scores were computed for each cell using predefined gene sets from the Molecular Signatures Database (MsigDB). Pearson’s correlation was calculated between GSVA scores and the primary trajectory component to evaluate the relationship between pathway activity and transcriptional progression. Gene expression trends for candidate early transformation markers were visualized along pseudotime. These analyses were used to characterize temporal dynamics of transcriptional reprogramming during the transition from tumor-initiating cells (TICs) to tumor stem-like (tSTM) cells.

### RNA Velocity Analysis

RNA velocity analysis was conducted using the VeloVAE framework to infer directional transcriptional dynamics and predict future cell state transitions. Spliced and unspliced transcript count matrices were generated using the Kallisto|Bustools pipeline with a pre-built human reference index optimized for RNA velocity inference. The resulting matrices were processed through VeloVAE, a variational autoencoder-based model that estimates latent time, kinetic parameters, and RNA velocity vectors across cells. Following standard preprocessing, dimensionality reduction and clustering were re-applied to ensure alignment between velocity-derived trajectories and existing cell annotations. The inferred velocity field and latent temporal ordering were used to assess lineage progression and validate pseudotime-based trajectories of tumor-initiating cell populations.

### RNA In Situ Hybridization

RNA fluorescence in situ hybridization (RNA-FISH) was performed to validate the spatial expression of early transformation markers in both normal and polyp. FFPE sections were cut at 5 μm thickness and mounted on Superfrost Plus glass slides. Sections were deparaffinized, subjected to heat-mediated antigen retrieval, and hybridized with RNA probes. ViewRNA^™^ Tissue Fluorescence Assay (Thermo Scientific, QVT0646B) was performed to detect RNA expression. RNA probes for *SOD3* and *MMP7* (VX06. Assay ID: VA1–3004554-VT and VA1–12258-VT) were obtained from Thermo Scientific. FISH assay was performed per the manufacturer’s protocol. Nuclei were counterstained with DAPI for cellular localization. Fluorescence imaging was conducted using a confocal microscope equipped with a 40× oil-immersion objective to assess marker expression patterns in situ. RNA-FISH was used to corroborate scRNA-seq–based identification of early transformation signatures in morphologically normal and polyp tissue compartments.

### Analysis of publicly available data

The QC-filtered data from the Colorectal Molecular Atlas Project [38] was downloaded from the HTAN data portal: https://data.humantumoratlas.org. For this study, processed Seurat object for discovery datasets were downloaded. All the downstream processing was performed according to the methods described in previous sections.

### Statistical Analysis

All analyses were performed in R (v4.1.0) unless otherwise specified. Differentially expressed genes (DEGs) were identified using the *FindAllMarkers* function in Seurat (v3.1.0), which applies the Wilcoxon rank-sum test with Benjamini–Hochberg correction for multiple testing. Genes were required to be expressed in at least 10% of cells in either cluster (*min.pct* = 0.1) and to show an absolute log fold change greater than 0.25 (*logfc.threshold* = 0.25). Genes with adjusted *p* < 0.05 and average log2 fold-change > 0.25 were considered significant. GSEA was performed using *clusterProfiler*, with significance defined as FDR-adjusted *q* < 0.05. GSVA scores were correlated with pseudotime using Pearson’s correlation. ROC analyses were performed using the pROC package. For each gene, area-under-the-curve (AUC), sensitivity, and specificity across thresholds were calculated, and the optimal threshold was determined by Youden’s index. Visualizations were performed with ggplot2 and Seurat functions. Thresholds and statistical metrics are reported in figures and supplementary tables.

## 3. Results

### Single-cell transcriptomic profiling identifies tumor-associated epithelial subpopulations

**Fig. 1 –:**
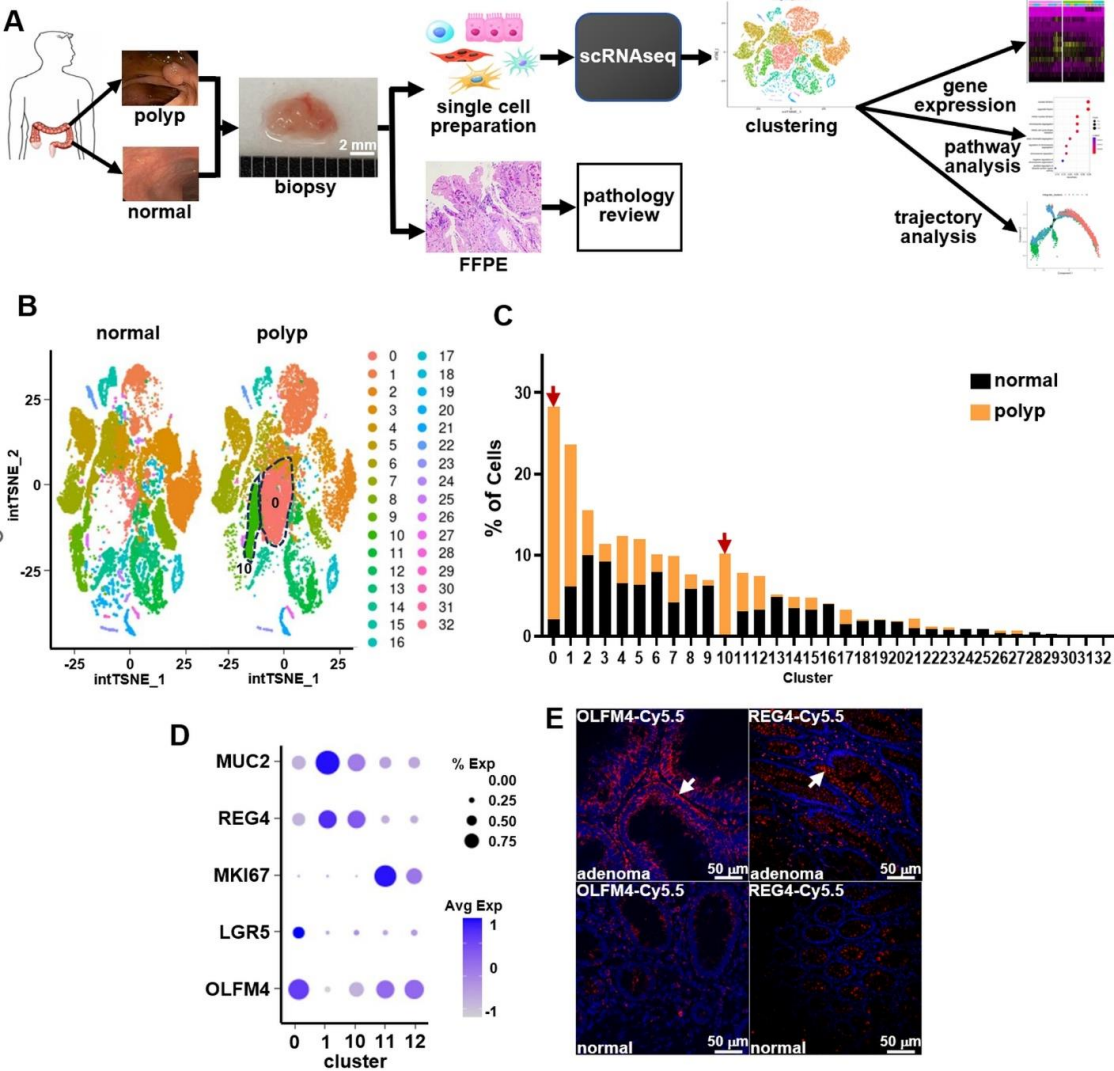
Single-cell transcriptomic profiling of human colon. **A**) Paired biopsies from 7 subjects were divided for scRNA-seq and pathology review. Polyps included premalignant lesions and one adenocarcinoma. **B**) Integration of 51,054 high-quality cells identified 33 transcriptionally distinct clusters with notable enrichment of clusters 0 and 10 in polyps. **C**) Frequency analysis confirmed tumor-specific enrichment of clusters 0 and 10. **D**) Dot plot revealed high expression of *OLFM4*, *LGR5*, *MKI67*, *REG4*, and *MUC2* in clusters 0 and 10, consistent with a proliferative, stem-like phenotype. **E**) Immunofluorescence staining validated upregulation of *OLFM4* and *REG4* in adenoma compared to minimal expression in matched normal tissue (arrows).

Single-cell RNA sequencing (scRNA-seq) was performed on paired colonic biopsies from 7 human subjects, and captured both polyps and adjacent histologically normal mucosa, Fig. 1A. The polyp cohort contained diverse histopathologic subtypes, including tubular adenomas, sessile and traditional serrated adenomas, and adenocarcinoma, as confirmed by pathology, Fig. S1, Table S1. After quality control, batch correction, and integration, Fig. S2A,B, 51,054 high-quality single-cell transcriptomes were obtained, including 31,376 normal and 19,678 polyp, Fig. 1B. After clustering, 33 transcriptionally distinct cell populations were identified. These clusters were annotated into major epithelial, stromal, and immune cell types and used to investigate tumor-associated transcriptional reprogramming, Table S3. Clusters 0 and 10 were markedly enriched in polyp-derived epithelial cells compared to normal, and defined tumor-associated subpopulations, Fig. 1B,C. Dot plot analysis revealed distinct transcriptional programs across clusters, Fig. 1D, Table S2, with cluster 0 exhibiting high expression of *OLFM4* and *LGR5*, consistent with a tumor-specific stem-like (tSTM) epithelial phenotype. Cluster 10 showed elevated *REG4* and *MUC2*, characteristic of a tumor-specific deep crypt secretory (tDCS) identity. In contrast, clusters 11 and 12 (normal stem-like, nSTM) and cluster 1 (normal deep crypt secretory, nDCS) were present in both normal and polyp tissues, reflecting homeostatic epithelial populations. Immunofluorescence validated strong upregulation of *OLFM4* and *REG4* in adenomatous epithelium relative to minimal expression in paired normal mucosa, Fig. 1E.

### Differential expression defines marker genes of tSTM and tDCS states

Comparative analysis revealed distinct transcriptional and pathway signatures between tumor-associated and normal epithelial populations, Fig. S3, Table S4. Tumor-specific stem-like cells (tSTM, cluster 0) exhibited strong upregulation of *OLFM4*, *LEFTY1*, *SOD3*, *LCN2*, *SLC12A2*, and *MMP7*, consistent with proliferative, inflammatory, and invasive phenotypes. Pathway enrichment confirmed activation of oncogenic and stress-related programs, including E2F targets, G2/M checkpoint, MYC signaling, KRAS signaling, TNFα/NFκB signaling, and EMT, and highlight a proliferative and mesenchymal-like state. Tumor-specific deep crypt secretory cells (tDCS, cluster 10) demonstrated elevated expression of *TFF1*, *FABP1*, *CKB*, *PRAP1*, *IL32*, *GPRC5A*, and *SOD3*, and were consistent with secretory lineage plasticity and immune or stress-associated signaling. These transcriptional and pathway programs distinguish tumor-enriched epithelial subtypes from homeostatic stem-like (nSTM, clusters 11 and 12) and deep crypt secretory (nDCS, cluster 1) populations, and underscore their roles in neoplastic progression.

### tSTM and tDCS cells activate distinct oncogenic programs

Gene set enrichment analysis (GSEA) revealed distinct pathway activation profiles in tumor-associated epithelial populations, Fig. S4. In tumor-specific stem-like (tSTM, cluster 0) cells, hallmark programs, including TNFα/NFκB signaling, epithelial–mesenchymal transition (EMT), KRAS signaling, E2F targets, and G2/M checkpoint, were significantly upregulated, consistent with a proliferative, inflammatory, and invasive transcriptional state. Tumor-specific deep crypt secretory (tDCS, cluster 10) cells were enriched for apical junction, apoptosis, EMT, MYC signaling, and E2F targets, reflecting secretory lineage reprogramming and stress-associated differentiation.

### Subclustering and lineage trajectory analyses identify TIC precursors of tSTM cells

**Fig. 2 –:**
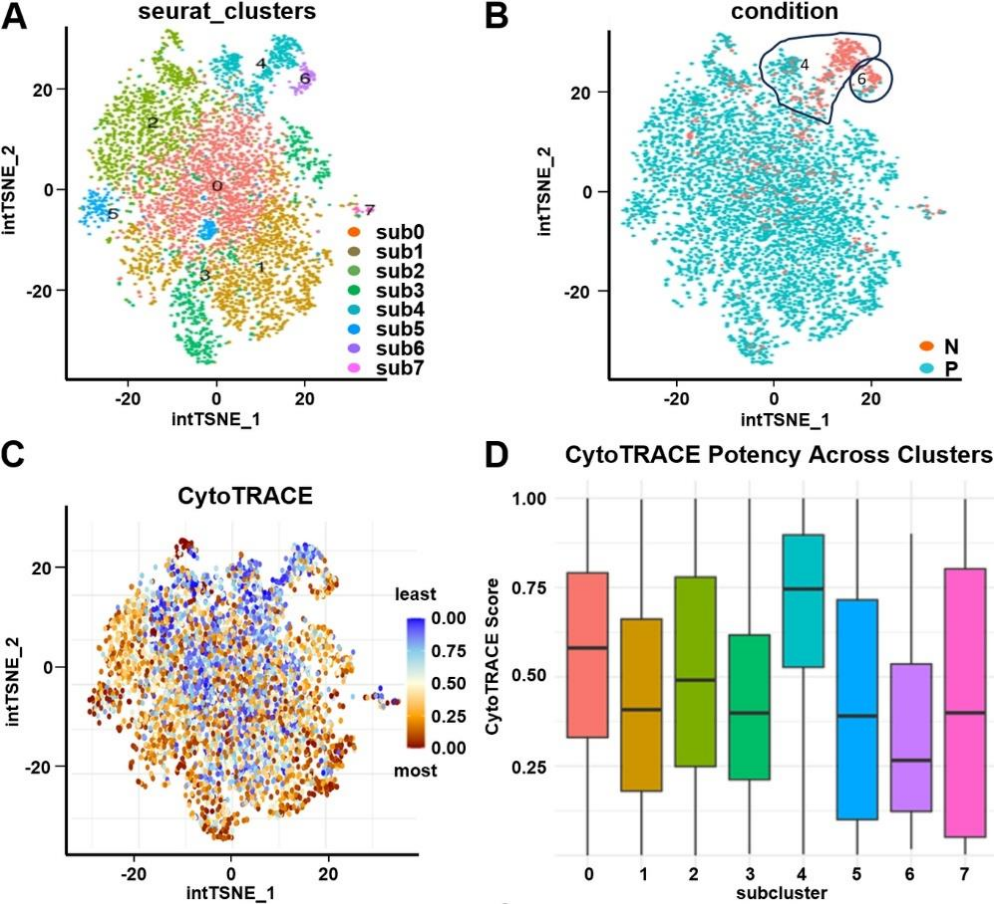
Identification of tumor initiating cells (TICs). **A**) t-SNE plot showed 8 transcriptionally distinct epithelial subclusters (sub0-sub7) identified by Seurat clustering of the tSTM population. **B**) t-SNE plot colored by sample condition show cells derived from normal (N, orange) and polyp (P, cyan). **C**) CytoTRACE analysis of tSTM population showed heterogeneity in differentiation states across the population. **D**) CytoTRACE potency analysis demonstrated highest stemness potential in sub4 with intermediate levels in sub6.

**Fig. 3 –:**
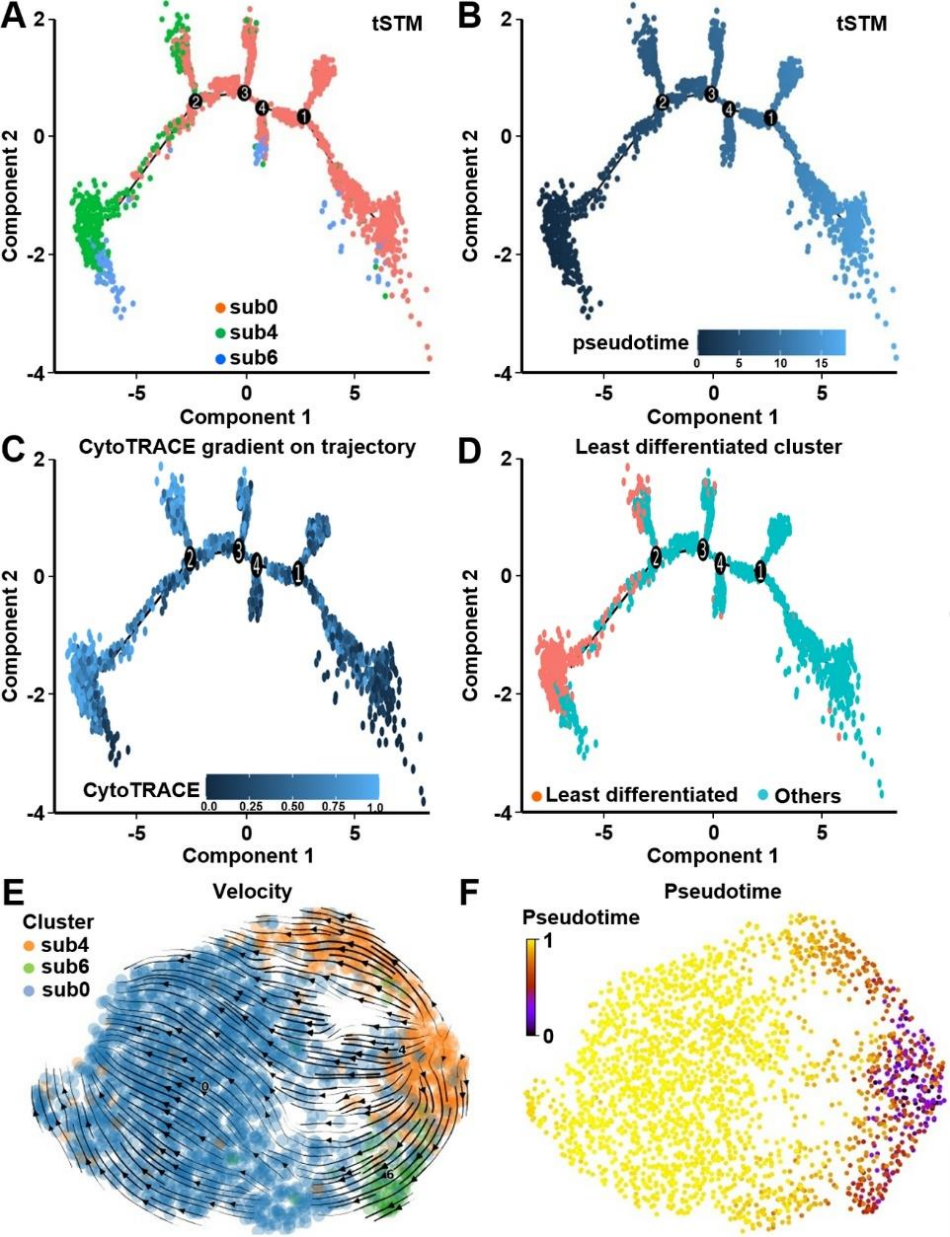
Trajectory inference and lineage progression of tSTM subclusters. **A**) Monocle 2 trajectory analysis revealed differentiation paths for subclusters 0, 4, and 6. Subclusters 4 and 6 are positioned at early stages, and subcluster 0 represents a polyp-enriched epithelial branch. **B**) Pseudotime ordering demonstrated a continuum from early to late states with terminal branches corresponding to more differentiated populations. **C**) CytoTRACE mapping along the trajectory confirmed the highest stemness scores at the trajectory root. **D**) Cells with the least differentiated state localized to the trajectory origin. **E**) RNA velocity analysis showed directional flow from subcluster 4 toward subclusters 6 and 0 to support a unidirectional differentiation pathway. **F**) Pseudotime heatmap further validated the temporal progression from early (purple) to late (yellow) states, consistent with RNA velocity and transcriptional activity.

To investigate the developmental origin of tumor-specific stem-like cells, tSTM (cluster 0) was further resolved into 8 epithelial subclusters, Fig. 2A. Among these, subclusters 4 and 6 were predominantly derived from histologically normal mucosa and identified as candidate tumor-initiating cells (TICs), whereas subcluster 0 was enriched in polyp tissue, Fig. 2B. Subcluster 4 exhibited the highest stemness potential using CytoTRACE analysis with intermediate levels in subclusters 6, Fig. 2C,D. Monocle 2 trajectory analysis of TIC (sub 4 and sub 6) and one of the tSTM clusters (sub 0) revealed a lineage continuum in which subclusters 4 and 6 mapped to early states and progressed directionally toward sub0, representing a polyp-enriched epithelial branch, Fig. 3A,B. Projection of CytoTRACE scores along pseudotime confirmed that cells at the trajectory root exhibited the greatest stem cell potential, Fig. 3C,D. RNA velocity analysis was performed to further support this model, and showed that transcriptional flow from subclusters 4 and 6 toward subcluster 0, consistent with a unidirectional differentiation pathway from early-stage TICs to tumor-specific stem-like states, Fig. 3E,F.

### CNV analysis reveals genomic instability in tumor-associated cells

Copy number variation (CNV) from the single-cell transcriptomes was evaluated using InferCNV to provide orthogonal evidence of neoplastic transformation. nSTM cells originating from normal epithelium were used as Reference. Epithelial clusters from normal mucosa showed minimal CNV alterations, consistent with genomic stability, Fig. S5A. Polyp-derived tSTM and nSTM populations from the observation clusters displayed widespread chromosomal amplifications and deletions, Fig. S5B. Notably, TICs (Cluster 0_N) did not exhibit significant chromosomal aberrations, supporting their origin from normal epithelium. These findings demonstrate that tumor-associated epithelial subtypes are defined not only by transcriptional and pathway reprogramming but also by underlying genomic instability, highlighting their central role in early neoplastic progression.

### Trajectory analysis reveals progressive transcriptional reprogramming

The movement of these clusters was traced along component 1, Fig. S6A. Along this continuum, EMT activity progressively increased while oxidative phosphorylation declined, Fig. S6B,C consistent with a transition toward mesenchymal-like and metabolically reprogrammed phenotypes. Gene set enrichment analysis (GSEA) of TICs revealed downregulation of oxidative phosphorylation and upregulation of EMT and interferon-α signaling pathways to indicate a shift toward a stress-responsive, pro-tumorigenic transcriptional program, Fig. S6D. The shift in cellular and metabolic pathways during evolution of cancer stem cells from TIC as evident from trajectory analysis, Fig. S6E,F.

### Early transformation markers revealed by pseudotime analysis and validated by RNA-FISH

**Fig. 4 –:**
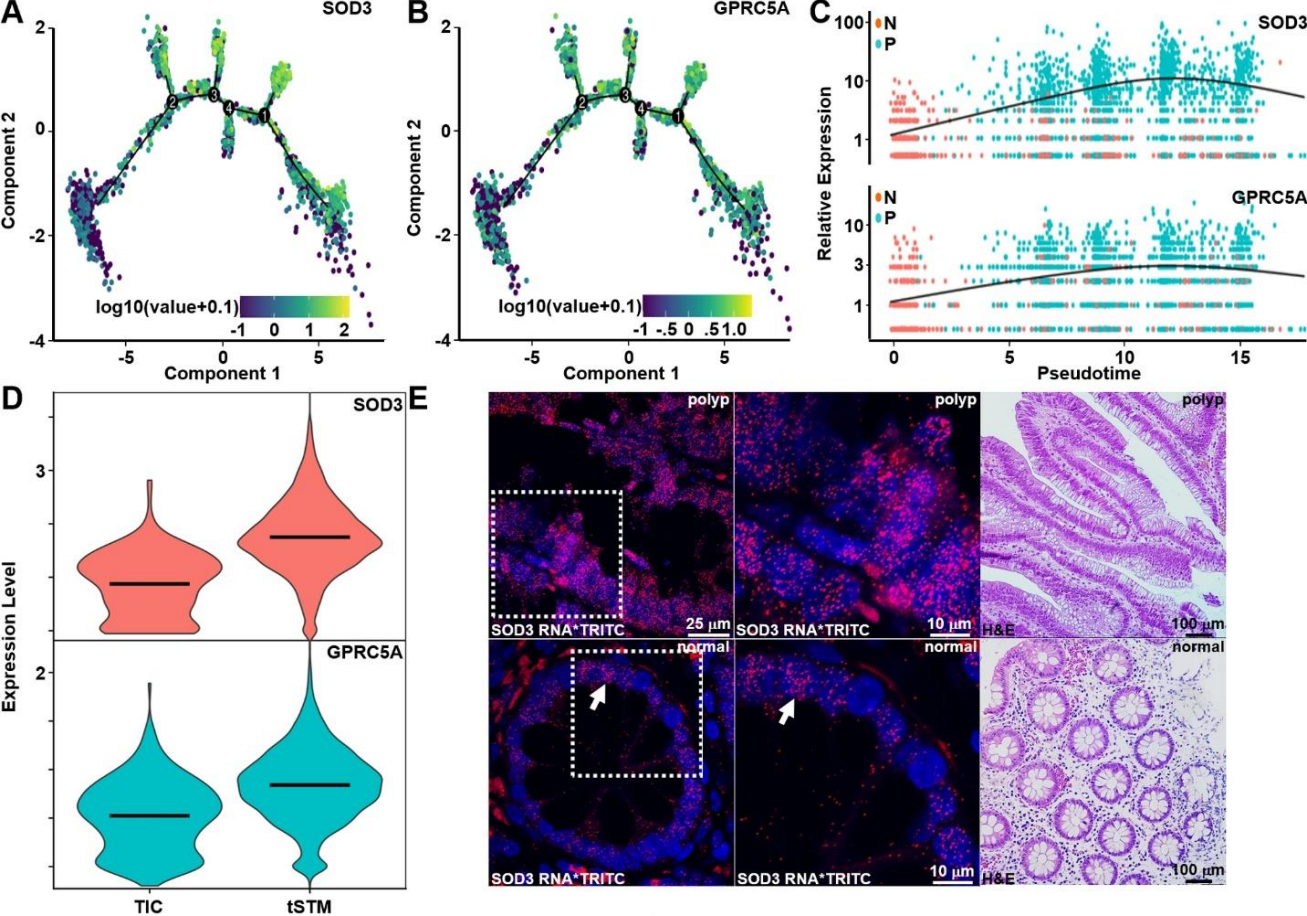
Progressive induction of *SOD3* and *GPRC5A* defines early neoplastic transition. **A**,**B**) Feature plots of the pseudotime trajectory highlight low expression of *SOD3* and *GPRC5A* in early states with progressive upregulation toward terminal branches, consistent with their role in epithelial transformation. **C**) Pseudotime scatter plots comparing cells from normal mucosa (N) and polyps (P) demonstrate increasing expression of both genes over time with greater induction in polyp-derived cells to support their involvement in polyp-associated state transitions. Smoothed regression curves (black lines) illustrate continuous expression dynamics. **D**) Violin plots show significantly elevated levels of *SOD3* and *GPRC5A* in tSTM cells compared to TICs to support their emergence as biomarkers of later transformation stages. **E**) RNA-FISH validation confirms upregulation of *SOD3* transcripts in polyp tissue relative to normal mucosa. Insets with higher magnification highlight strong epithelial staining in polyps (arrows), while minimal signal is observed in normal crypts. TRITC (red) marks RNA puncta, DAPI (blue) shows nuclei, and adjacent H&E-stained sections provide histological context.

Expression of early transformation markers, including *SOD3*, and *GPRC5A*, rose steadily along pseudotime, and highlight their role as candidate molecular indicators of neoplastic progression, Fig. 4A–C. The expression of *SOD3* and *GPRC5A* was found to be significantly higher in tSTM compared to TIC, Fig. 4D. RNA-FISH analysis confirmed spatial upregulation of *SOD3* in adenomatous crypt with minimal expression in adjacent normal mucosa, Fig. 4E. specimen.

### TIC-specific biomarkers identified and validated by statistical and spatial analyses

**Fig. 5 –:**
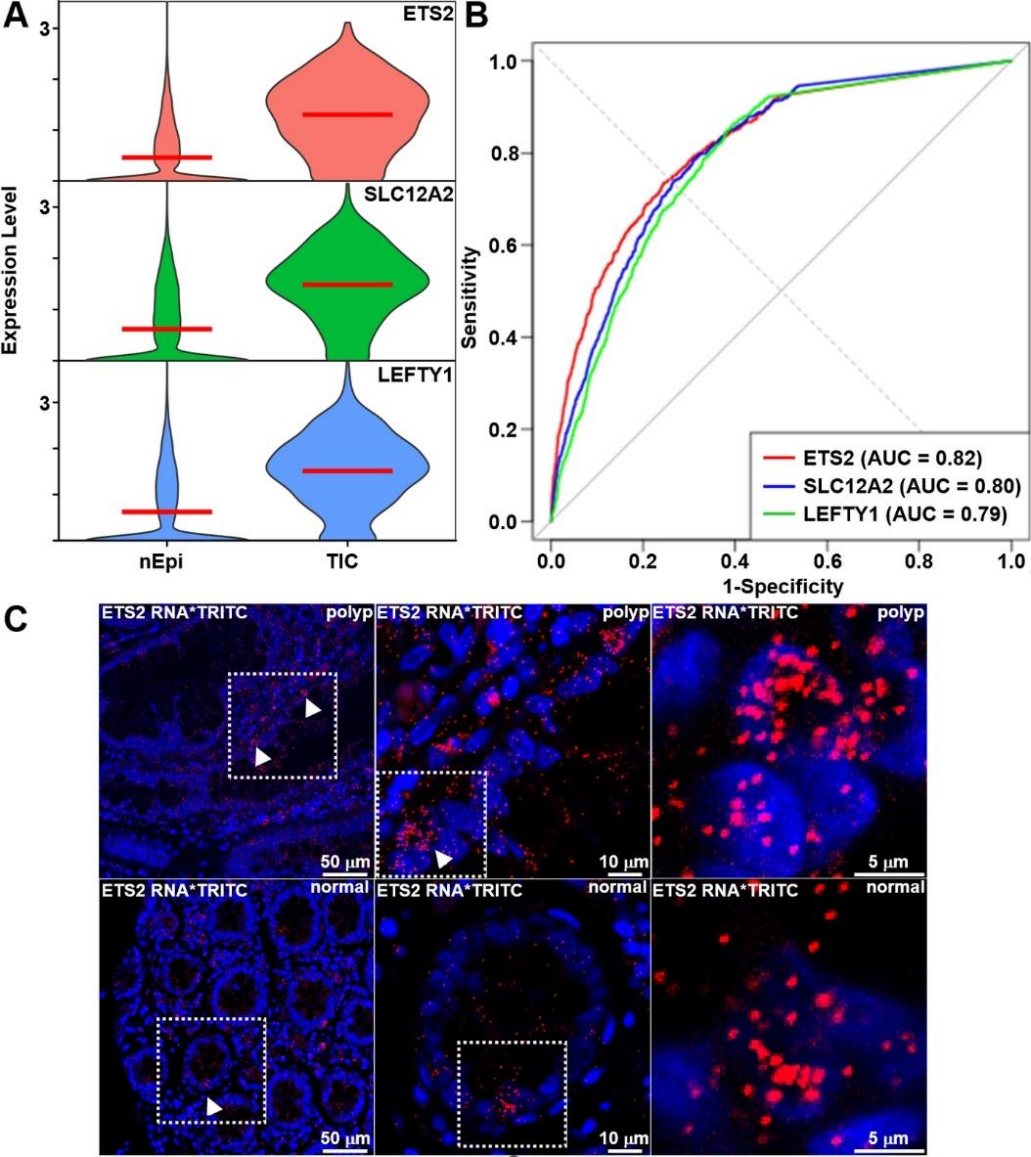
Identification of TIC specific biomarkers. **A**) Violin plots show significantly higher expression of *ETS2*, *SLC12A2*, and *LEFTY1* in TICs compared to normal epithelial cells (nEpi). **B**) ROC curves demonstrate diagnostic potential, with AUCs of 0.82 (*ETS2*), 0.80 (*SLC12A2*), and 0.79 (*LEFTY1*). Sensitivity/specificity values were 0.735/0.747 for *ETS2*, 0.786/0.691 for *SLC12A2*, and 0.862/0.604 for *LEFTY1*. Differential expression analysis confirmed robust upregulation of ETS2 (avg_log2FC = 1.98, adj. p = 6.48E-181), SLC12A2 (avg_log2FC = 1.62, adj. p = 1.77E-158), and LEFTY1 (avg_log2FC = 1.55, adj. p = 4.11E-150) in TICs. **C**) RNA-FISH validation of *ETS2* revealed increased transcript signal in TIC cells (arrowheads) compared to adjacent epithelial cells in polyp and normal specimen. TRITC (red) marks RNA puncta, nuclei are counterstained with DAPI (blue), and high-magnification insets highlight increased *ETS2* expression in polyp epithelium.

TIC specific markers were also identified. Since, TIC were identified in cluster 0 originating from normal specimen. A differential gene expression analysis was performed on normal epithelial cells. Comparative analysis of TICs versus normal epithelial cells revealed strong enrichment of *ETS2*, *SLC12A2*, and *LEFTY1* within TIC populations, Fig. 5A,B. Each gene demonstrated high diagnostic performance in distinguishing TICs from normal epithelium with sensitivities ranging from 0.73 to 0.86. Spatial validation by RNA-FISH confirmed the presence of TICs in normal crypt epithelium marked by elevated *ETS2* transcript levels, Fig. 5C. Notably, TICs were also identified in polyp specimen, Fig. 5C.

### Independent validation of TICs using publicly available datasets

**Fig. 6 –:**
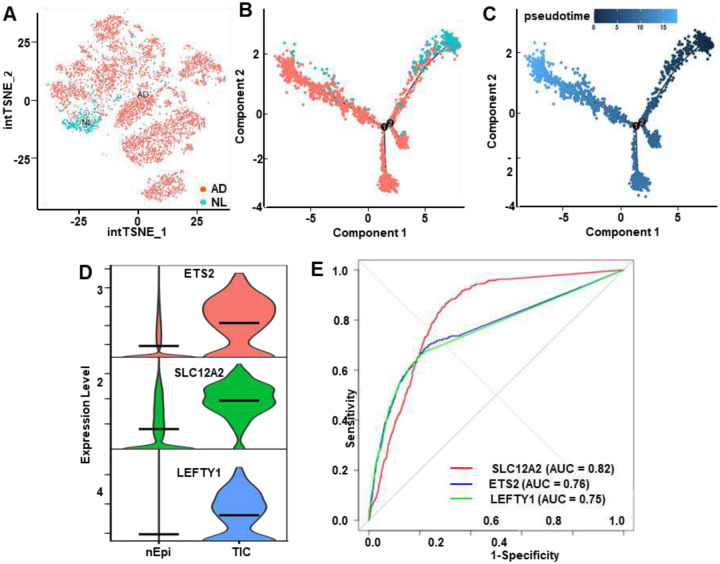
Independent dataset validates TIC-associated biomarkers. **A**) Sub-clustering of COLON MAP cells identified adenoma-associated (AD) populations and a subset originating from normal (NL) specimen. **B,C**) Trajectory and pseudotime analyses showed that AD cells arise from TIC-like populations and progress along a developmental continuum from early root states to terminal differentiated branches. **D**) Violin plots confirmed elevated expression of *ETS2*, *SLC12A2*, and *LEFTY1* in TICs compared to normal epithelial cells (nEpi). **E**) ROC curve analysis demonstrated diagnostic performance of these candidate genes with AUCs of 0.82 (*SLC12A2*), 0.76 (*ETS2*), and 0.75 (*LEFTY1*). Differential expression analysis supported significant upregulation of *ETS2* (log2FC = 2.82, adj. p = 1.78E-97, sensitivity = 0.690, specificity = 0.777), *SLC12A2* (log2FC = 2.79, adj. p = 6.07E-125, sensitivity = 0.868, specificity = 0.696), and *LEFTY1* (log2FC = 2.99, adj. p = 3.47E-120, sensitivity = 0.657, specificity = 0.804).

An independent dataset was analyzed to further validate presence of TICs in normal epithelium [38]. Chen et. al, 2021 reported that tubular adenomas are enriched in cells referred to as ASC (Adenoma-Specific Cells) and expressed *OLFM4* and *LGR5* [38]. Subclustering of ASCs from COLON MAP study identified a subset of cells originating from normal specimens, Fig. 6A. These cells were designated as TICs. On single cell trajectory, TIC cells appear to be the origin of the ASCs, Fig. 6B,C. The TIC markers *ETS2*, *SLC12A2* and *LEFTY1* showed higher expression on TICs in comparison with normal epithelium, Fig. 6D. Statistical analysis showed high sensitivity and specificity for the detection of TICs for these genes, Fig. 6E.

## 4. Discussion

Here, we applied single-cell transcriptomic profiling (scRNA-seq) to paired biopsies of colonic adenomas and adjacent normal mucosa from 7 patients to investigate the earliest cellular and molecular events in colorectal tumorigenesis. This analysis uncovered 2 tumor-associated epithelial populations, including a tumor-specific stem-like (tSTM, cluster 0) state defined by *OLFM4* and *LGR5*, and a tumor-specific deep crypt secretory (tDCS, cluster 10) state characterized by *REG4* with limited *MUC2*. Subclustering of the tSTM population revealed 8 epithelial subsets, among which subclusters 4 and 6, largely derived from histologically normal mucosa, emerged as candidate tumor-initiating cells (TICs). CytoTRACE, pseudotime ordering, and RNA velocity consistently placed TICs at the root of lineage trajectories progressing toward polyp-enriched tSTM cells, supporting their role as precursors of neoplastic stem-like states.

The transition from TICs to tSTM cells was defined by progressive transcriptional reprogramming, including activation of epithelial–mesenchymal transition (EMT) and suppression of oxidative phosphorylation, signaling a shift toward a mesenchymal-like, metabolically reprogrammed phenotype. GSVA and GSEA analyses further revealed enrichment of proliferative and inflammatory pathways, including E2F, MYC, KRAS, TNFα/NFκB, and stress-associated signaling. Copy number variation (CNV) profiling provided orthogonal evidence of genomic instability in polyp STM cells relative to their normal counterparts.

Along this continuum, pseudotime analysis identified *SOD3* and *GPRC5A* as early transformation markers with progressive upregulation, validated by RNA-FISH in adenomatous crypts. Comparative analyses of TICs versus normal epithelium identified *ETS2*, *SLC12A2*, and *LEFTY1* as robust TIC-specific biomarkers with high diagnostic sensitivity and specificity, findings independently validated using the COLON MAP dataset [38]. These results establish TICs as the developmental origin of neoplastic stem-like states, implicate EMT and metabolic reprogramming as central drivers of early transformation, and nominate biomarkers with translational potential for early CRC detection.

Previous expression studies in CRC have largely focused on advanced tumors. Here, we identify TICs and marker genes differentially expressed within histologically normal and premalignant colonic epithelium, implicating TICs as a potential initiating population for CRC. These cells may serve as novel targets for prevention, early detection, and therapy, and could also underlie therapeutic resistance and relapse [39–41]. EMT, upregulated in tSTM cells by GSEA of Hallmark pathways, has long been linked to CSC function, metastasis, and recurrence [39–41]. Cells undergoing EMT can acquire stem-like properties [42,43], reflecting the plasticity by which epithelial cells adopt mesenchymal phenotypes with enhanced migratory and invasive potential [44]. This transition facilitates asymmetric self-renewal and maintenance of undifferentiated progenitors while generating differentiated progeny [45]. In the context of CRC, EMT may drive the emergence of TICs, consistent with our observation that tSTM cells show greater EMT activity than TICs.

Distinct epithelial subpopulations were further organized into stem-like and secretory lineages. The tDCS cells expressed *REG4* and *MUC2* which are the markers of deep crypt secretory (DCS) cells [46]. DCS cells are known to support the stem cell niche through secretion of Notch and EGF ligands [47]. In adenomas, *REG4*^+^ DCS cells were enriched alongside *OLFM4*^+^ CSCs, suggesting that tDCS cells may provide trophic support to *OLFM4*^+^ stem-like populations. This finding aligns with previous studies linking *OLFM4* to stem cell clusters in adenomas and patient-derived organoids [48].

Finally, this study sheds light on early molecular events in CRC initiation. The patient cohort in present study was relatively small, with only 7 patients, which restricts statistical power and generalizability across adenoma subtypes. Although limited by sample size, our findings were validated using an independent COLON MAP dataset of 63 specimens, including 30 tumors [38]. The COLON MAP study reported that tubular adenomas arise from WNT-driven expansion of stem cells [38]. In the present study, 80% of tSTMs were derived from tubular adenoma specimens supporting the notion that tubular adenomas originate through stem cell expansion. Similar to our results, adenoma-specific stem cells were shown to originate from TIC-like populations expressing *ETS2*, *SLC12A2*, and *LEFTY1*. As a cross-sectional analysis, the proposed progression from normal stem-like cells through TICs to tumor-specific states is inferred rather than directly observed. Lineage relationships were reconstructed computationally using CytoTRACE, pseudotime, and RNA velocity, which provide strong correlative evidence, but would be stronger with experimental validation in organoid or xenograft models. scRNA-seq reduces spatial resolution, and although selected markers were validated by RNA-FISH and immunofluorescence, broader proteomic and spatial validation would strengthen the findings. A multi-faceted experimental approach will be essential to fully define the role of TICs in colorectal cancer initiation.

## Conclusion

scRNA-seq was used to perform an in-silico analysis of the gene expression profiles from individual epithelial cells in paired pre-malignant (adenoma) and adjacent normal colonic mucosa obtained from patients at increased risk for CRC. The study identified tumor initiating cells which express ETS2, SLC12A2 and LEFTY1. SOD3, and GPRC5A were identified as early marker genes expressed in evolving tumor stem cells. The EMT transition pathway plays a key role in transforming normal into cancer stem cells. These TICs may be promising targets for prevention, early detection, and therapy of CRC.

## Supplementary Material

1

Supplementary Files

This is a list of supplementary files associated with this preprint. Click to download.

- TableS1.xlsx
- TableS2.csv
- TableS3.xlsx
- TableS4.xlsx

## Data Availability

The raw transcriptome data will be deposited in the Genome Sequence Archive (GSA). All other relevant data are available on request from the authors. The code used to generate the graphic presentation is available on GitHub (https://github.com/tstephie/Jaiswal_scRNA-seq_colon).

## Abbreviations:

AUC: Area Under the Curve
CRC: Colorectal Cancer
CSC: Cancer Stem Cell
DCS: Deep Crypt Secretory
DEG: Differentially Expressed Gene
EMT: Epithelial-to-Mesenchymal Transition
FFPE: Formalin-Fixed Paraffin-Embedded
FISH (RNA-FISH): RNA Fluorescence In Situ Hybridization
GO: Gene Ontology
GSEA: Gene Set Enrichment Analysis
GS: Gene Signature
GSVA: Gene Set Variation Analysis
H&E: Hematoxylin & Eosin
MSigDB: Molecular Signatures Database
nDCS: Normal Deep Crypt Secretory
NES: Normalized Enrichment Score
nSTM: Normal Stem
PCA: Principal Component Analysis
PHOS: Oxidative Phosphorylation
R: R Statistical Computing Environment
RNA-FISH: RNA Fluorescence In Situ Hybridization
scRNA-seq: single cell RNA sequencing
STM: Stem
tDCS: Tumor-Specific Deep Crypt Secretory
TIC: Tumor Initiating Cell
t-SNE: t-distributed Stochastic Neighbor Embedding
tSTM: Tumor-Specific Stem
UMAP: Uniform Manifold Approximation and Projection
UMI: Unique Molecular Identifier
