# Supplementary material for: Identification of tumor initiating cells and early marker genes in normal colonic epithelium that lead to neoplastic transformation
Source: Res Sq. 2025 Oct 27:rs.3.rs-7914753. Preprint. [Version 1] doi: 10.21203/rs.3.rs-7914753/v1 (PMC12636725; doi:10.21203/rs.3.rs-7914753/v1)
Supplement: 1 [file NIHPPRS7914753V1-supplement-1.pdf]

Supplementary Figures

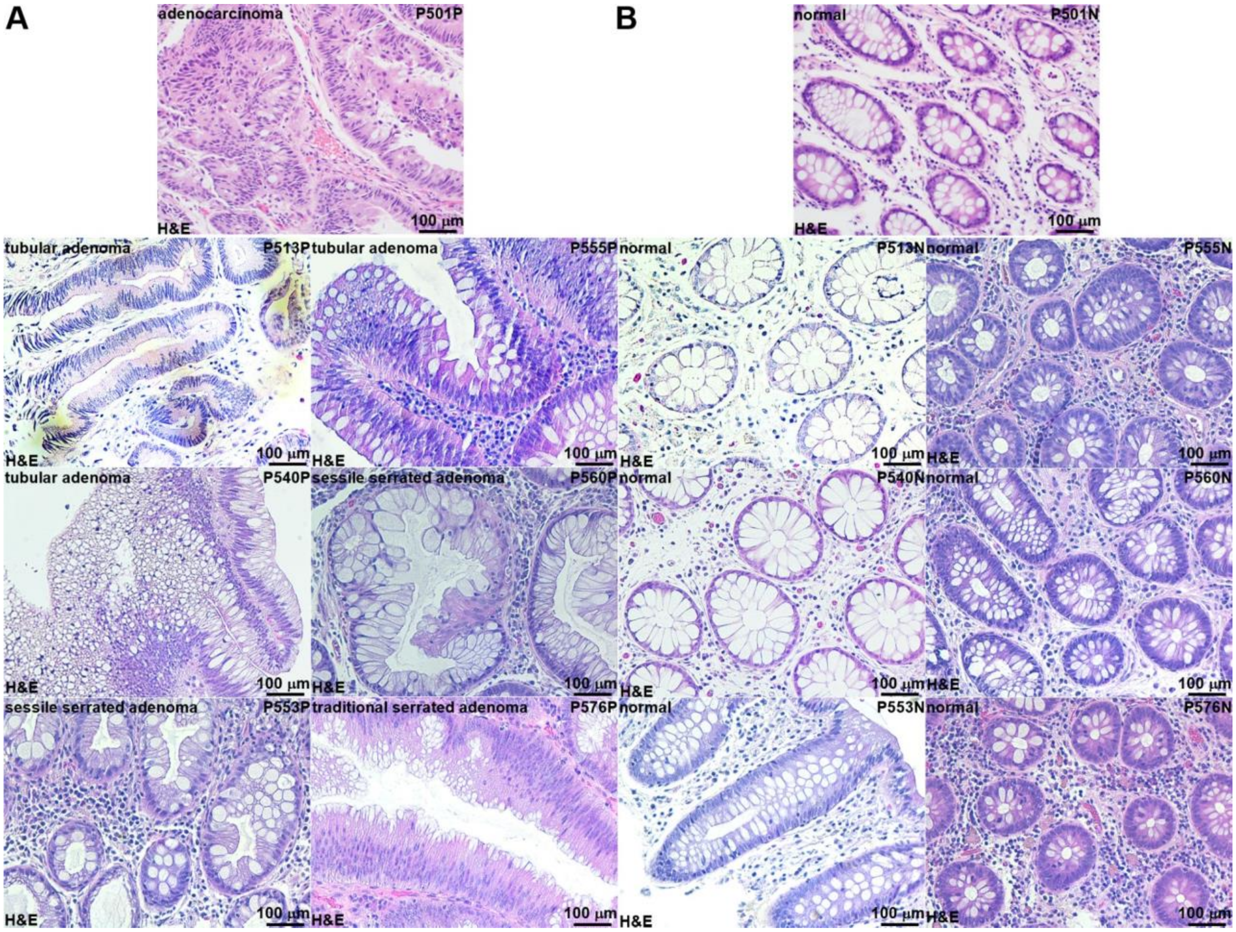

Fig. **S1 – Histopathologic features of polyps and matched normal colonic mucosa**. **A**) H&E-stained sections of colonic polyps from 7 patients illustrate diverse histologic subtypes, including tubular adenomas (P513P, P555P, P540P), sessile serrated adenomas (P553P, P560P), traditional serrated adenoma (P576P), and adenocarcinoma (P501P). These images demonstrate the morphological heterogeneity of neoplastic lesions subjected to scRNA-seq, with features such as pseudostratified columnar epithelium, serrated crypts, mucin depletion, and variable nuclear atypia and dysplasia. **B**) H&E-stained sections of paired histologically normal colonic mucosa from the same patients (P501N, P513N, P540N, P553N, P555N, P560N, P576N) display intact crypt architecture, uniform epithelial morphology, and absence of dysplasia. These matched controls provide a histologic baseline for comparative single-cell transcriptomic profiling and enable identification of neoplastic transformation–associated molecular changes.

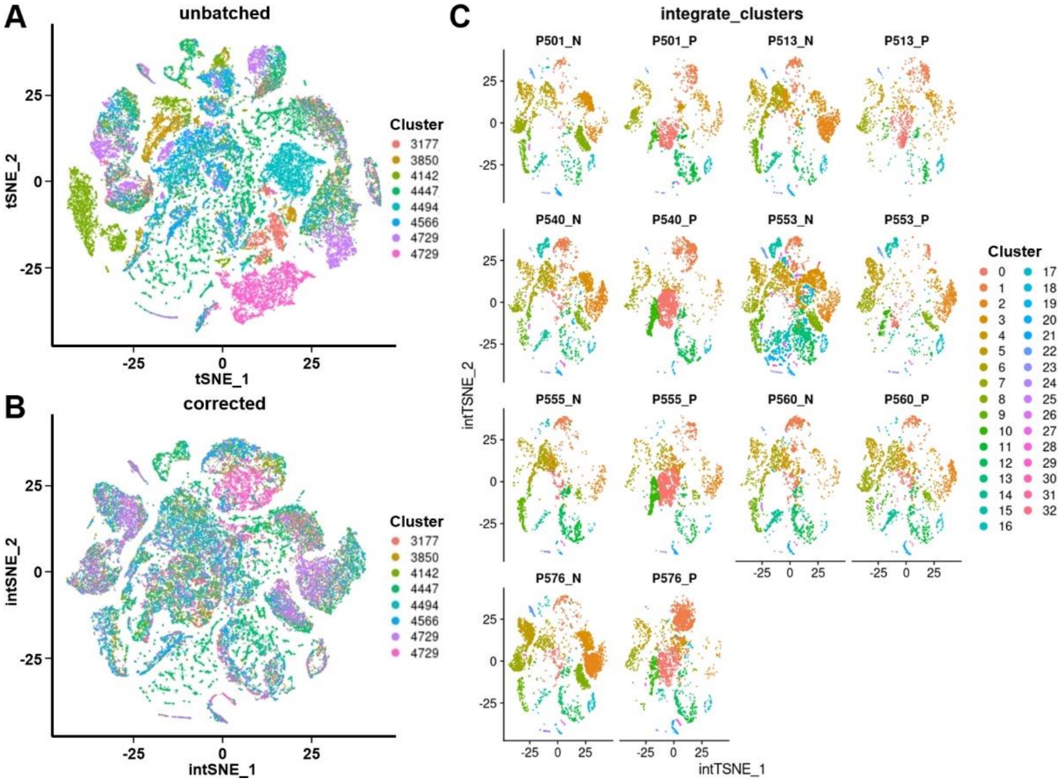

Fig. **S2 – Batch correction and integration of scRNA-seq data**. **A**) t-SNE plot of uncorrected single-cell transcriptomes from all samples prior to batch integration shows strong batch effects and patient-specific separation that obscures the underlying biological variation. **B**) Batch correction with the Seurat integration pipeline successfully reduced technical artifacts, and allows cells from different patients to intermingle within shared clusters to improve alignment of biologically similar populations. **C**) t-SNE plots of integrated clusters from each patient sample (P501, P513, P540, P553, P555, P560, P576) show paired normal (N) and polyp (P) specimens. Each point represents a single cell, colored by its assigned cluster (0–32), and demonstrates consistent cluster distribution across patients after integration. These results confirm that downstream clustering reflects true biological variation rather than technical noise.

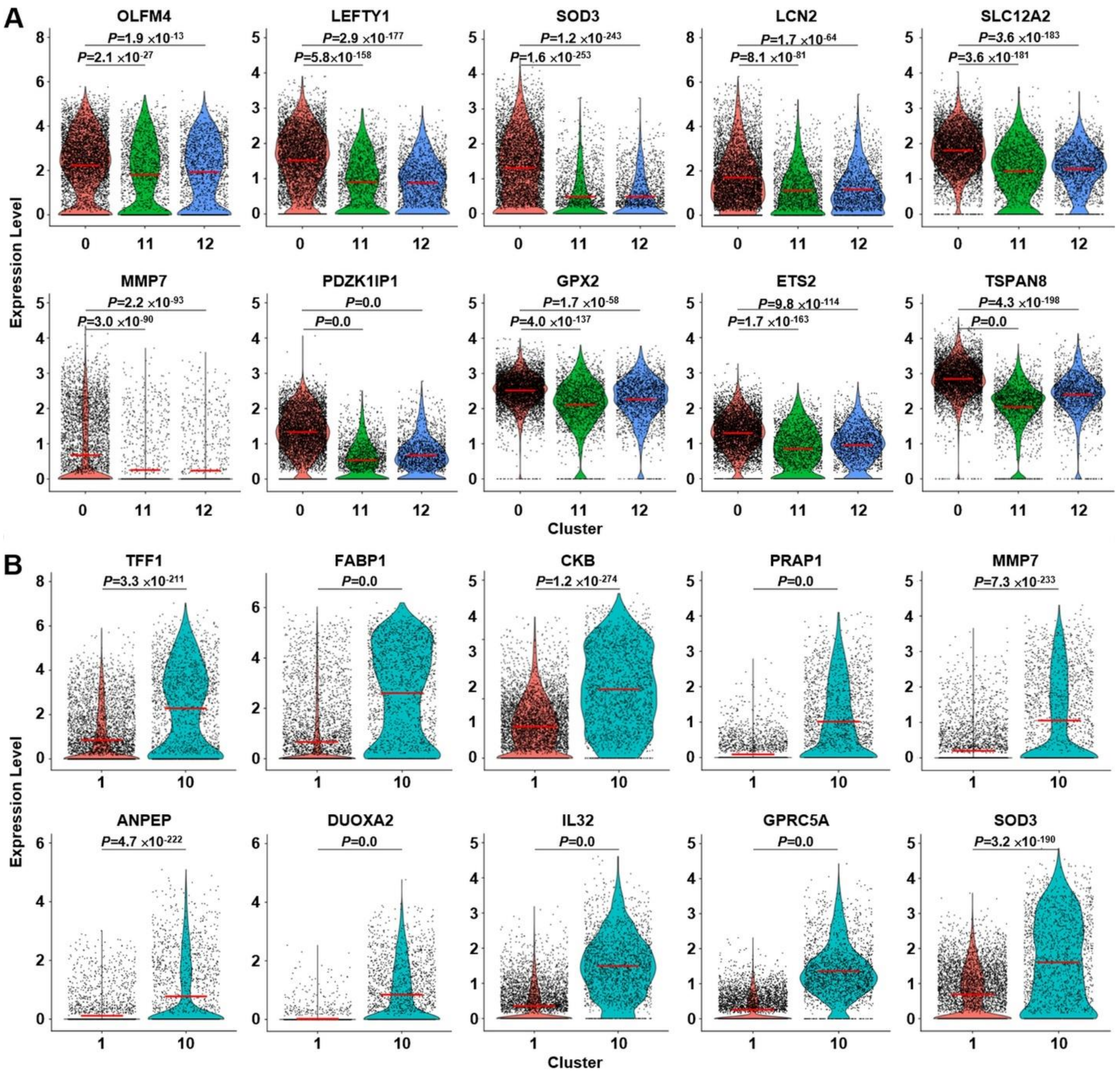

Fig. S3 – Differential gene expression in tumor-associated epithelial clusters. **A**) Violin plots show significant upregulation of multiple genes in tumor-specific stem-like (tSTM, cluster 0) cells compared to normal stem-like populations (nTSM, clusters 11 and 12). Markers include *OLFM4*, *LEFTY1*, *LCN2*, *SOD3*, *SLC12A2*, *GPX2*, *ETS2*, and *TSPAN8*, as well as *MMP7*, a metalloproteinase associated with invasion and extracellular matrix remodeling. These signatures indicate proliferative, regenerative, and pro-inflammatory states characteristic of tumorigenesis. **B**) Tumor-specific deep crypt secretory (tDCS, cluster 10) cells also exhibited elevated expression of *TFF1*, *FABP1*, *CKB*, *PRAP1*, *MMP7*, *ANPEP*, *DUOXA2*, *IL32*, *GPRC5A*, and *SOD3*, to highlight distinct secretory and stress-associated programs. Each violin plot depicts cell-level expression (dots), distribution density, and mean expression level (red line).

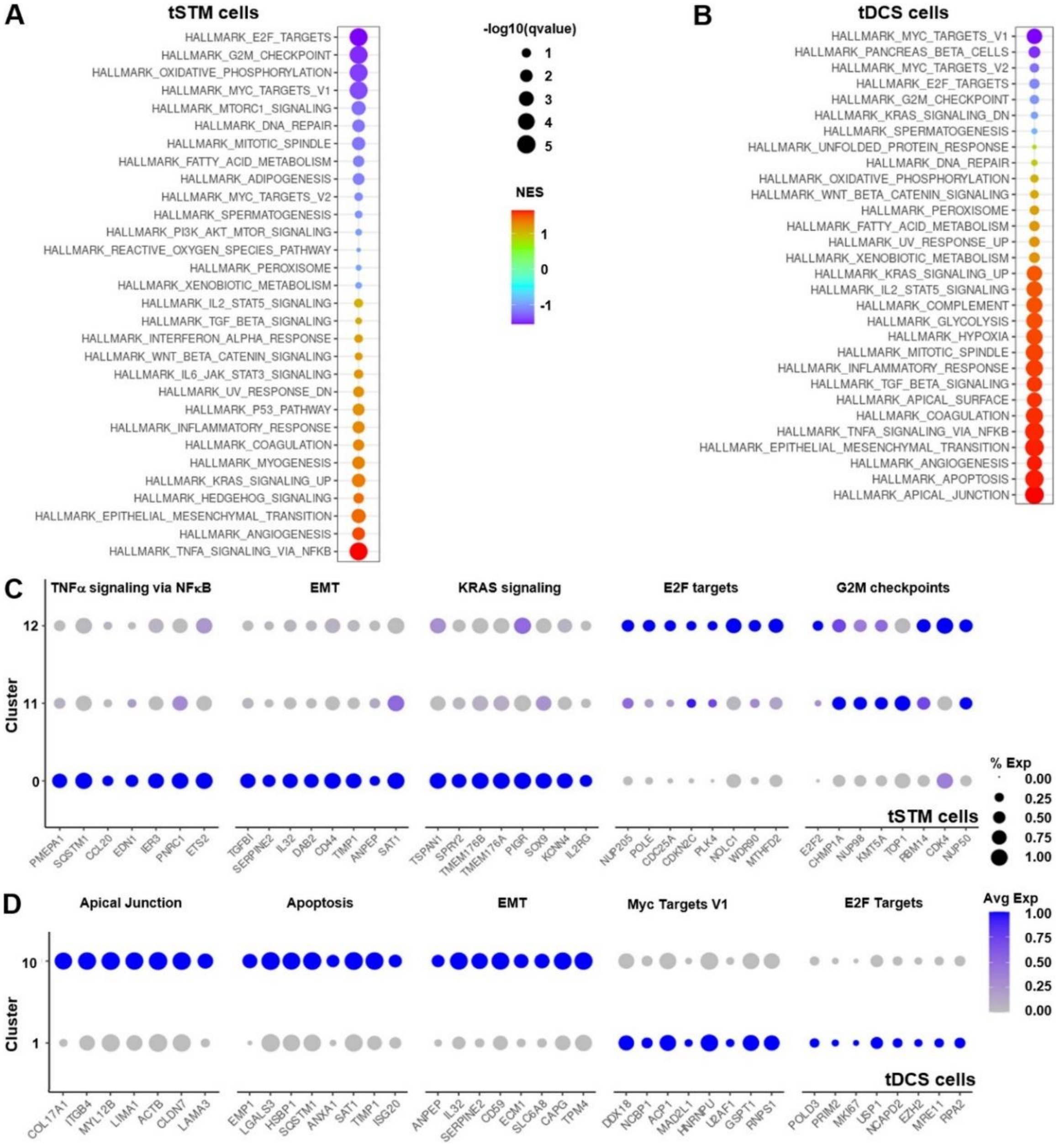

Fig. S4 – Pathway enrichment analysis of tumor-associated epithelial clusters. **A**) Cluster 0 (tSTM cells) showed strong enrichment of oncogenic and inflammatory programs, including E2F targets, G2/M checkpoint, MYC targets, KRAS signaling, TNFα/NFκB signaling, and EMT, consistent with a proliferative and invasive state. **B**) Cluster 10 (tDCS cells) was enriched for pathways involving MYC signaling, G2/M checkpoint, apoptosis, apical junctions, and pancreatic beta cell signatures, indicating partial lineage reprogramming and secretory differentiation. Pathway enrichment is visualized by normalized enrichment score (NES, color scale) and statistical significance (dot size, $-\log_{10}$ adjusted q-value). **C**) In tSTM cells, canonical pathway genes were highly expressed, including *NFKBIA*, *RELB* (TNFα/NFκB), *VIM*, *FN1* (EMT), *DUSP4*, *RHOB* (KRAS), and *CCNB1*, *MKI67* (cell cycle), highlighting proliferative, pro-inflammatory, and mesenchymal-like features. **D**) In tDCS cells, hallmark programs such as apical junction, apoptosis, EMT, and MYC/E2F targets were supported by upregulation of junctional genes (*TJP1*, *CLDN4*), pro-apoptotic regulators (*PMAIP1*, *BAX*), and cell cycle markers (*PCNA*, *CDC20*), and underscores a distinct secretory and stress-associated phenotype.

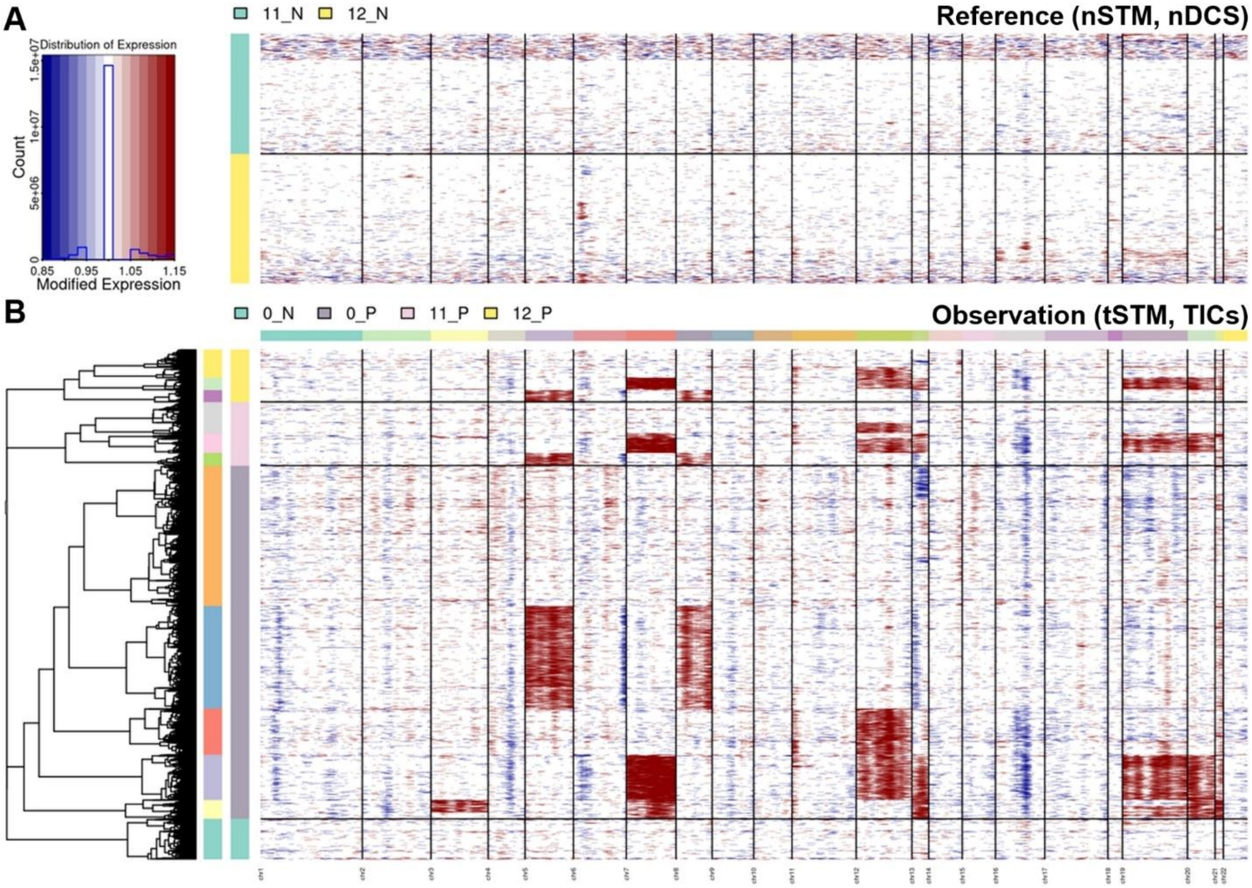

Fig. S5 – CNV analysis of normal- and polyp-derived epithelial populations. inferCNV heatmap displays gene-level CNVs inferred from single-cell transcriptomes. Reference cells from normal stem cell clusters (clusters 11_N and 12_N) show minimal CNV patterns, consistent with genomic stability. In contrast, observation cells from stem cells populations derived from polyps (clusters O_P, 11_P and 12_P) exhibit widespread chromosomal alterations, with regions of relative amplification (red) and deletion (blue). The TIC (cluster 0_N) population did not show significant chromosomal aberration. These CNV profiles provide orthogonal evidence confirming the malignant state of polyp-associated epithelial populations compared to their normal counterparts.

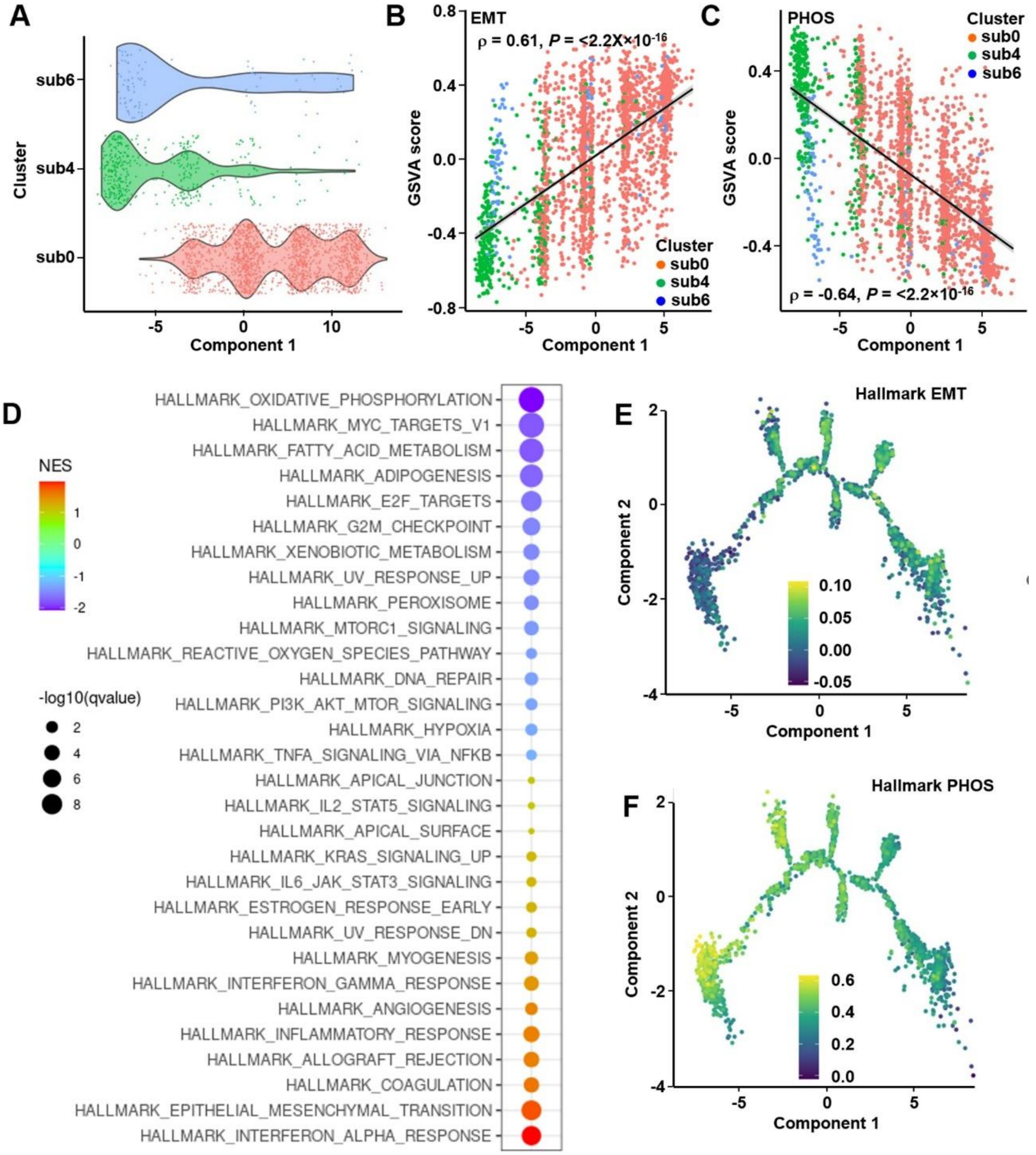

Fig. **S6 – Pathway dynamics during TIC-to-tSTM transitions**. **A**) Violin plots show the distribution of cells from tSTM subclusters (sub0, sub4, sub6) along the pseudotime trajectory, and demonstrates progressive transcriptional shifts across these populations. **B**,**C**) GSVA scores plotted against component 1 highlight pathway trade-offs. EMT scores increase significantly ($\rho = 0.61$, $P < 2.2\times10^{-16}$), whereas oxidative phosphorylation (PHOS) scores decrease ($\rho = -0.64$, $P < 2.2\times10^{-16}$), and indicate a balance between metabolic reprogramming and mesenchymal-like transformation. **D**) GSEA comparing TIC subclusters (sub4, sub6) and tSTM (sub0) identified significant pathway differences, including oxidative phosphorylation downregulation and activation of EMT and oncogenic programs. **E**,**F**) GSVA scores projected along pseudotime further validated these dynamic state transitions, showing progressive activation of EMT and suppression of oxidative phosphorylation during the TIC-to-tSTM trajectory.
